# Evaluation of the Possible Mechanisms of Antihypertensive Activity of *Loranthus micranthus*: An African Mistletoe

**DOI:** 10.1155/2011/159439

**Published:** 2011-09-12

**Authors:** Bamidele A. Iwalokun, Sedoten A. Hodonu, Stella Nwoke, Olabisi Ojo, Phillip U. Agomo

**Affiliations:** ^1^Department of Biochemistry and Nutrition, Nigerian Institute of Medical Research, PMB 2013, Yaba, Lagos, Nigeria; ^2^Department of Biological Sciences, Boise State University, Boise, ID 83725-1320, USA; ^3^Department of Biochemistry, College of Medicine University of Lagos, PMB 12003, Idi-Araba, Lagos, Nigeria; ^4^Department of Microbiology and Molecular Genetics, University of Pittsburgh, Pittsburgh, PA 15269, USA

## Abstract

*Loranthus micranthus (LM)*, also called African mistletoe is a major Nigerian *Loranthaceae* plant used traditionally to treat hypertension. The methanolic leaf extract of the plant (LMME) has been shown to elicit anti-hypertensive activity in rats but mechanism remains unclear. This study was undertaken to study the effect of LM on pressor-induced contraction of rat aorta smooth muscles and serum lipid profiles in mice. The LMME was partitioned to produce n-butanol (NBF-LMME), chloroform (CF-LMME), ethyl acetate (EAF-LMME) and water (WF-LMME) fractions. The median effective concentrations and maximum relaxation of the fractions were determined against epinephrine and KCl pre-contracted rat aorta ring model. Serum lipid profiles and nitric oxide (NO) were determined spectrophotometrically in mice administered per orally 250 mg/kg b.w. of each fraction for 21 days. Data were analyzed statistically. NBF-LMME elicited the highest dose-dependent inhibitory effect on rat aorta pre-contracted with norepinephrine and KCl, followed in decreasing order by WF-LMME > CF-LMME > EAF-LMME. Similar order of activity was observed in the ability of these fractions to inhibit elevation in artherogenic lipids, raise serum nitric oxide and reduce cardiac arginase in mice. We conclude the anti-hypertensive activity of *L. micranthus* involve anti-artherogenic events, vasorelaxation, cardiac arginase reduction and NO elevation.

## 1. Introduction

Hypertension, classically defined as systolic pressure >165 mmHg or diastolic blood pressure >95 mmHg or both in adult is a noninfectious disease of global dimension in prevalence, incidence, complications, and deaths [1, 2]. Deaths due to hypertension arise from cerebrovascular and cardiovascular complications such as stroke, end-stage renal disease, congestive heart failure, myocardial infarction, and cardiac arrest [2]. Early detection and commencement of chemotherapy are essential in preventing or delaying these complications and enhancing survival of the afflicted patients [3]. To treat hypertension coupled with associated complications, use has been made of drugs derived from plants. They include digitoxin from *Digitalis purpurea *(foxglove), reserpine from *Rauwolfia serpentina* (snakeroot), aspirin from *Salix alba* (willow bark), tetramethylpyrazine, also known as Ligustrazine from *Jatropha podagrica,* and tetrandrine from *Stephenia tetradra* [4, 5]. These plant-derived pharmaceuticals have scientifically been proven to elicit antihypertensive activity via multiple mechanisms. These mechanisms are elicited to counteract the effect of hypertension and associated risk factors such as hypercholesterolemia, hypertriglyceridaemia, and oxidative stress on blood vessel walls [6]. They include direct vasodilation of the blood vessel, blocking of calcium channels, inhibition of *α*-adrenoreceptor response, induction of negative ionotropic response of smooth muscle, inhibition of platelet aggregation, reduction of vascular resistance, and improvement of pulmonary oxygen utilization [7–9]. Enhanced activity of nitric oxide and improved handling of intracellular calcium has also been found to play a critical role in the reduction of vascular resistance and blood pressure that are elevated in hypertensive rats and humans [10, 11]. In the last 2 decades, plants have remained historically important as sources of novel compounds with potentials of being channeled into drug pipelines for the development of safe, efficacious, and cost-effective antihypertensive drugs. In sub-Saharan Africa, the initial ethnopharmacological surveys have identified over 100 species of plants with antihypertensive activity in animals and humans [12, 13]. *Loranthus micranthus* from the *Loranthaceae* family is one of these plants. The plant is a semiparasitic shrub that grows by obtaining nutrients and support from a host of trees such *Kola acuminata, K. nitida, Mangifera indica, Azadirachta indica, Jatropha curcas,* and* Persia* sp. in Nigeria [14]. The plant has for many years been documented both in Nigeria and South Africa to be a traditional medicine of high repute in the treatment of diabetes and hypertension, as a remedy against schizophrenia and an immune booster [15, 16]. A study by Obatomi et al. [17] revealed hypotensive effect of *L. micranthus* on spontaneously induced hypertensive rats. Other *Loranthaceae* plants that have been validated to be hypotensive in function include *L. ferruginea, L. yadoriki *and* L. tanakee* [18–20]. In our recent work, we found the methanolic leaf extract of* L. micranthus* to elicit a dose-dependent inhibition of blood pressure elevation in adrenaline induced hypertensive rat (Iwalokun et al., unpublished) to corroborate previous reports [15, 17]. Recently, Ameer et al. [21] reported the n-butanol fraction from the methanolic leaf extract of *L. ferrugineus* was found to elicit dose-dependent inhibition of KCl and phenylephrine induced aortic ring contraction coupled with blood pressure lowering effect in rats. Therefore, we hypothesize that mechanisms exist to mediate the hypotensive action of *L. micranthus*, which remain largely unknown. Meanwhile, the use of *L. micranthus* in animals has been found to be safe with an LD50 >5000 mg/kg [16] and without adverse biochemical effects in rats [22]. Aqueous and methanolic leaf extracts of *L. micranthus* also contain tannins, terpenoids, flavonoids, and alkaloids phytoconstituents, which have been implicated for the various pharmacological activities of the plant including antibacterial and antidiabetic properties [14–16, 23, 24]. Interestingly, these phytoconstituents were also found in other *Loranthaceae *plants in association with antihypertensive activity [18–20]. Given the long-term use of *L. micranthus* as an antihypertensive plant, it is undoubtedly important to understand its mechanisms of action in order to advance its potential as a source of novel compounds for future development of antihypertensive drugs. In this study, the vascular effects of different fractions obtained by solvent-solvent fractionation of crude methanolic leaf extract of *L. micranthus *were investigated using isolated rat aortic ring preparation under KCl and epinephrine induction. The effect of chronic administration of these fractions, including the crude methanolic extract on blood lipid profiles, and nitric oxide levels were also studied.

## 2. Materials and Methods

### 2.1. Plant Material

Aerial part of the plant *Loranthus micranthus* was harvested from a Kolanut tree (*Kola acuminata*) from Sagamu, Ogun State, during the beginning of rainy season in March, 2009. The plant was taken to the Department of Botany, University of Lagos, Nigeria for authentication. After authentication, a voucher sample of the plant (no. 0184) was deposited in the herbarium of the University.

### 2.2. Preparation of *Loranthus micranthus* Methanolic Leaf Extracts (LMME) and Its Fractions

The leaves of *Loranthus micranthus* were rinsed in distilled water to remove dirt, dried in an air-oven at 40°C for 3 days, and then pulverized into fine powder that passed through a 30-mesh sieve. The ground plant material (100 g) was subsequently extracted with 1000 mL of 80% methanol using Soxhlet apparatus. The resulting crude methanolic extract was filtered by passage through a Whatmann no. 3 filter paper followed by concentration in vacuo at 40°C using a rotary evaporator and freeze drying. The yield of the freeze-dried sample representing LMME was calculated to be 10.3%. For the preparation of LMME fractions, 10 g of the freeze-dried sample was suspended in 150 mL deionized water and mixed thoroughly. The mixture was transferred to a 1L-separating funnel for sequential fractionation by solvent-solvent extraction method with sequential addition of chloroform (4 × 100 mL), ethylacetate (4 × 100 mL), and n-butanol (4 × 100 mL). The resulting fractions collected into separate conical flasks were concentrated in vacuo and subsequently freeze-dried to obtain 3.3 g, 1.4 g, 2.9 g, and 2.4 g of chloroform (CF-LMME), ethylacetate (EAF-LMME), n-butanol (NBF-LMME), and water (WF-LMME) fractions, respectively. Another portion of the ground plant material (50 g), extracted with methanol, fractionated, and freeze-dried as described previously was submitted to Phytochemical screening according to Sofowora [25], Trease and Evans [26], and Harborne [27]. Standard solutions of saponin, nicotine, quercetin, and tannic acid (10 mg/mL each) from Sigma (USA) were prepared fresh as qualitative standards for saponins, alkaloids, flavonoids and tannin, respectively. Positive reactions were qualitatively graded as mildly present (+), moderately present (++), and highly present (+++) based on color intensity.

### 2.3. Animals and Chronic Administration of LMME Fractions

Albino mice weighing between 21–23 g (mean weight) and rats (90–105 g, mean weight = 97.4 g) obtained from the animal Facility of the Nigerian Institute of Medical Research (NIMR), Yaba-Lagos, Nigeria were used for the *in vivo *and *in vitro* experiments, respectively. Experimentation was carried out according to the National Institute of Health (USA) guidelines on the care and use of laboratory animals for experiments [28], while the study protocol was approved by the Animal Committee of the National Institute of Medical Laboratory Sciences, Nigeria. The animals were individually housed in stainless metabolic cages under standard environmental conditions of temperature (26 ± 1°C) and humidity (60%–65%). They were placed on 12 h light-dark cycle and fed with standard rodent diets (Ladoke Feeds, Nigeria) with deionized water *ad libitum*. The animals were acclimatized in this manner for 7 days prior to use for experimentation. On the 8th day, the acclimatized mice were fasted for 10 h but with access to water. Two mice were randomly selected and sacrificed after a light chloroform anaesthesia to collect whole blood by cardiac puncture into plain tubes for baseline determination of lipid, nitric oxide and creatinine levels. In a fasted state, the mice were randomized into 5 groups of 7 mice per group. Animals in groups I–IV were administered per orally with 250 mg/kg of body weight doses each of CF-LMME, EAF-LMME, NBF-LMME, and WF-LMME (dissolved in 200 uL of normal saline) daily (10.00-11.00 h) for 21 days using an oral cannula. Animals in group V received the vehicle (i.e., 200 uL of normal saline (0.85% NaCl)) in parallel with the extract treated mice. The animals had free access to feed and deionized water throughout the experimental period.

### 2.4. Biochemical Assays

On day 22, after a 10 h fast but with access to deionized water, the animals in groups 1-V were sacrificed by cervical dislocation after light chloroform anaesthesia. Blood samples collected from each animal by cardiac puncture into plain tubes were allowed to clot and tubes were subsequently centrifuged at 2000 rpm for 5 min to obtain sera that were transferred into new tubes and kept at *‒*20°C until used for bioassays. The heart tissue was immediately excised, rinsed in deionised water, blotted dry between filter paper, and weighed. The weighed tissue was homogenized in 3 mL of ice-cold Tris-HCl buffer (pH 7.5) containing 0.08 M MnCl_2_ for preactivation of cardiac arginase using a glass homogenizer. After a preincubation for 30 min, cardiac arginase was assayed as described by Gayer and Dabich [29] using arginine (pH 9.5) at 37°C as a substrate. This method was based on the colorimetric determination at 490 nm of released urea nitrogen following reaction with 2,3-butanedione. One unit of enzyme releases one micromole of urea per minute under the assay conditions. Enzyme activity was expressed as units/g tissue. Serum total cholesterol was assayed as described by Siedel et al. [30], while the protocols of T. Gordon and M. Gordon [31] and Jacobs and VanDenmark [32] were adopted for the determination of HDL-cholesterol and triglycerides (TAG). LDL-cholesterol level was determined by calculation using the Friedwald formula [33] as follows:

(1) $$\begin{array}{cc} & \text{LDL-cholesterol}\\ & \;=\text{Total}\;\text{cholesterol}\;\left(\text{TC}\right)-\frac{\text{TAG}}{5}-\text{HDL-cholesterol}. \end{array}$$

Serum creatinine and total protein were determined using the alkaline picrate reagent [34] and biuret method [35]. Serum nitric oxide level was determined indirectly as its metabolic products (nitrate + nitrite ions) spectrophotometrically using a test kit (Boeringher, USA) in which all the nitrate ions in serum were first reduced to nitrite ions by nitrate reductase followed by the reaction between nitrite ions and the Greiss reagent (0.1% naphthylethylenediamine dihydrochloride in distilled water and 1% sulfanilamide in 5% H_3_PO_4_) to form a blue color solution [36]. Absorbance measurement was done at 540 nm against the reagent blank in which the serum sample was replaced with de-ionized water. The levels of nitric oxide in the experimental animals and control were determined by extrapolation from absorbance-concentration curve of the sodium nitrate standard solution (10–100 *μ*M).

### 2.5. Isolated Rat Aorta Ring Experiment

In this *in vitro* experiment, the acclimatized rats were each anaesthetized intraperitoneally with phenobarbitone sodium (60 mg/kg body weight), bled and exsanguinated. A midline incision was made through the sternum to open up the thoracic cavity and excise the aorta. Each aorta was sectioned into 4 rings of 3.5 mm long devoid of fat and connective tissue. The rings were placed horizontally in a tissue bath 10 mL of Kreb's-Ringer-Bicarbonate (KRB) buffer (pH 7.4) (118.2 mM NaCl, 4.7 mM KCl, 2.5 mM CaCl_2_·2H_2_0, 1.2 mM MgSO_4_, 1.2 mM KH_2_PO_4_, 11.7 mM glucose, and 25 mM NaHCO_3_) under 5% CO_2_ and 95% O_2_ atmosphere at 37°C. The aorta rings were maintained under a 1.5 g tension and allowed to equilibrate for 30 min prior to contraction induction with KCl and norepinephrine. The tension was supplied by a force-displacement transducer (model FT-03) that was coupled with a data acquisition system and a computer (AD Instrument, Sydney, Australia) and was connected to the tissue bath. During the equilibration period, two changes with fresh KRB (10 mL each) in the tissue bath were made to protect the aorta rings from toxicity that may arise from metabolic waste products. Contraction of the aorta ring smooth muscle was induced in separate experiments with 1 uM and 80 mM of norepinephrine and KCl, respectively. In each experiment, precontraction of the aorta ring was followed by cumulative addition of 0–1.5 mg/mL each of the LMME fractions: NBF-LMME, WF-LMME, CF-LMME, and EAF-LMME to observe their smooth muscle relaxant effects. Each fraction was added after attaining a stable relation response to a previous fraction at a time that ranged from 4–9 min. 

Each freeze-dried fraction was reconstituted by dissolution in Kreb's Ringer Bicarbonate buffer (pH 7.4) and diluted serially (2-fold) with the same buffer. Each fraction was assayed in triplicates at each tested concentration. The tension attained following contraction induction with norepinephrine and KCl and additions of LMME fractions was monitored and recorded. Relaxation, a measure of inhibition of contraction in aorta ring precontracted with either norepinephrine or KCl was measured in percentage and calculated as follows:

(2) $$\text{Relaxation}\;\left(R_{\max\;}\right),\;\text{\%}=\left(\frac{\text{Tc}-\text{Tt}}{\text{Tc}}\right)\times 100,$$

where Tc and Tt were tension due to KCl or norepinephrine (negative control) and an LMME fraction (test). EC_50_ the median effective concentration was defined as the concentration of the fraction required to inhibit aorta contraction by 50% of its precontracted state and was obtained by extrapolation from the relaxation-dose curve.

### 2.6. Statistical Analysis

Data were entered into Microsoft Excel 2007 and analysis was done using STATA statistical software version 11.1 (Statacorp, USA). Data were expressed as mean ± SEM (standard error mean) and percentages (%). Differences in mean values of biochemical parameters investigated between the treatment groups and the control and involving treatment period were analyzed using Duncan Multiple range test [37]. Relationship between variables such as serum total cholesterol and nitric oxide was evaluated by univariate regression analysis. Outcomes with *P* value below 0.05 were considered to be significant.

## 3. Results

**Figure 1:**
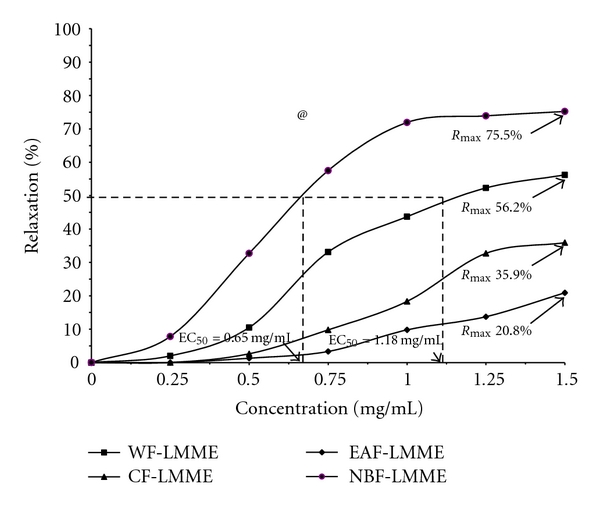
Median inhibitory concentration of and relaxation response to *Loranthus micranthus* methanolic extract fractions (LMME) by rat aorta rings precontracted with norepinephrine. WF-LMME = Water fraction; NBF-LMME = n-butanol fraction; CF-LMME = chloroform fraction; EAF-LMME = Ethyl acetate fraction. ^@^ *P* = 0.005, Mulvariate test of mean *R*_max_ values of the four fractions.

Induction of contraction of rat aorta ring with 1 uM norepinephrine and 80 mM KCl was observed to result in elevation of the baseline tension from 1.5 g to 1.53 ± 0.07 g and 1.56 ± 0.03 g, respectively (results not shown). The n-butanol fraction of the *L. micranthus *methanolic extract (i.e., NBF-LMME) elicited the highest concentration dependent inhibitory effect on aorta ring contraction due to norepinephrine with an EC_50_ of 0.65 mg/mL and maximum smooth muscle relaxation (*R*_max_) of 75.2% (Figure 1).

**Figure 2:**
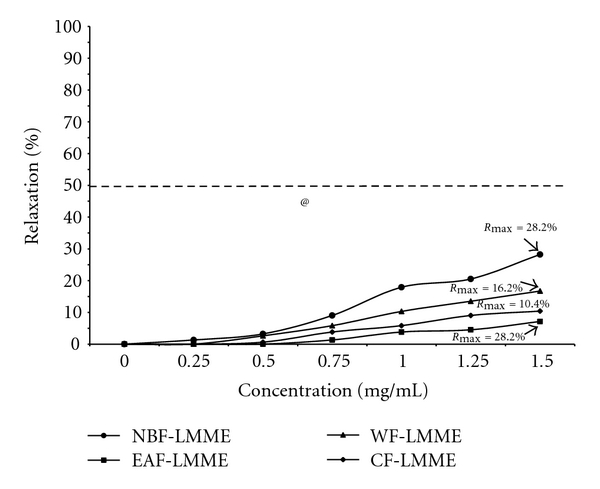
Median inhibitory concentration of and relaxation response to *Loranthus micranthus* methanolic extract (LMME) fractions by rat aorta rings precontracted with KCl. WF-LMME = Water fraction; NBF-LMME = n-butanol fraction; CF-LMME = Chlororform fraction; EAF-LMME = ethyl acetate fraction ^@^ *P* = 0.004 Multivariate test of mean *R*_max_ values of the four fractions.

This was followed 6 by the water fraction (i.e., WFLMME) with an EC50 of 1.18 mg/mL and *R*_max_ of 56.2% (Figure 1). The *R*_max_ values for CF-LMME and EAFLMME were 35.9% and 20.8% with both fractions eliciting EC50 >1.5 mg/mL (Figure 1), the maximum tested concentration for each fraction. On the whole, the disparity in the *R*_max_ values was significant (*P* = 0.005). The inhibitory effects of these fractions on KCl-induced aorta contraction also followed the same order but characterized by lower *R*_max_ values of 7.1%–28.2% and all fractions eliciting EC50 *>*1.5 mg/mL (Figure 2 ). On the whole, the disparity in the *R*_max_ values was significant (*P* = 0.004).

Changes in serum lipid levels in mice administered per orally 250 mg/kg body weight each of the LMME fractions for 21 days are shown in Table 1. Compared to baseline levels (day 0), very mild-to-moderate elevation in total cholesterol levels by 3.4%–7.4% and LDL-cholesterol levels by 30.2–37.4% that were significantly (*P* < 0.05) lower than the 28.6% and 131.9% respective increases in controls, were found in mice treated with NBF-LMME and WF-LMME. On day 21, significant (*P* < 0.05) reduction in TAG level (138.5 ± 3.4 versus 152.8 ± 0.6 mg/dL) compared to the control and by 6% when compared with baseline level 147.3 ± 2.5 mg/dL) was found in NBF-LMME treated mice. The disparity observed in HDL-cholesterol levels between day 0 and day 21 in control and LMME fractions-treated mice was not significant (41.3 ± 0.9 versus 40.6–41.6 ± 0.2–0.7 mg/dL; *P* > 0.05). 

The baseline serum total protein level (5.14 ± 0.05 g/dL) increased by 1.6% in the control and varied by 1.2%–1.7% after 21 days in mice treated with the LMME fractions, while serum creatinine elicited alterations that ranged from −8.8% to 17.6% in the treatment groups (Table 2). These alterations were not significantly different (*P* > 0.05) between the treatment groups and when compared with the control. However, the NBF-LMME-treated mice elicited 55% increase in baseline nitric oxide level (14.9 ± 0.1 umole/L) that was significantly higher than 3.7% increase observed in the control and 2.7%–22.1% increase found in other LMME fraction-treatment mice (Table 2). Overall, the decreasing order of potency of the fractions in reducing serum total cholesterol and triglyceride levels and increasing nitric oxide level after 21 days compared with the control levels was NBF-LMME > WF-LMME > CF-LMME > EAF-LMME. The NBF-LMME fraction also elicited the highest reduction in cardiac arginase activity by 11.7%, followed by WF-LMME (8.3%) (Table 2).

**Figure 3:**
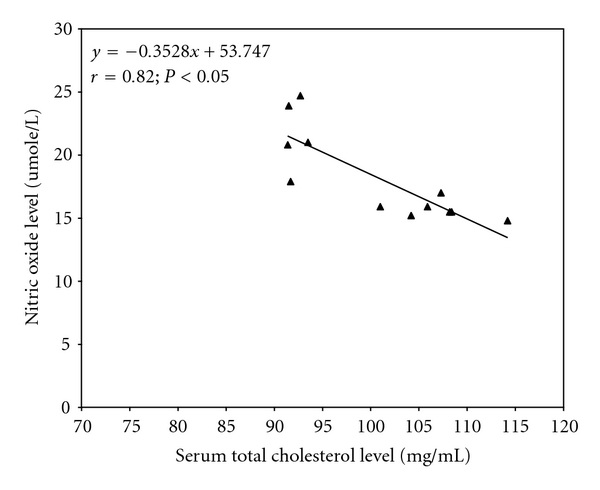
Regression analysis showing the relationship between serum total cholesterol and nitric oxide levels in LMMe-fraction treated mice for 21 days. Every 1 mg/dL decrease in serum total cholesterol was associated with 0.35 umole increase in NO.

**Figure 4:**
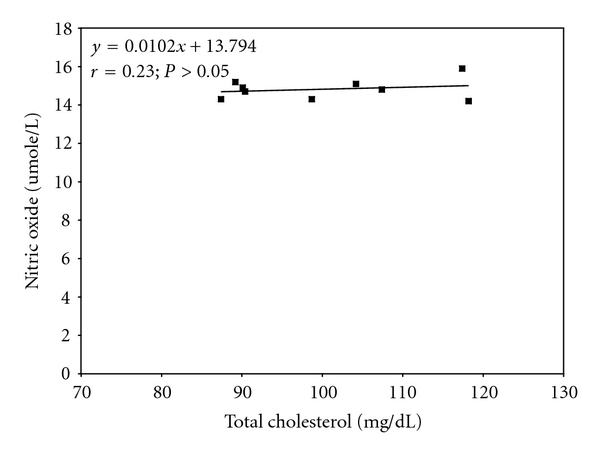
Regression analysis showing the relationship between serum total cholesterol and nitric oxide in the control mice.

Further analysis indicated that serum total cholesterol elicited a significant (*P* < 0.05) inverse correlation with serum nitric oxide level in mice administered the various fractions of *L. micranthus* methanolic extract for 21 days such that a decrease in serum total cholesterol by 1 mg/dL would result in an increase in serum nitric oxide by 0.35 umole/L (Figure 3). This relationship was not found in the control (Figure 4).

Phytochemical screening of the fractions summarized in Table 3 showed that high and moderate levels of terpenoids and steroids were present in the NBF-LMME fraction alone, while tannins, reducing sugars, and phenolics generally were in moderate abundance in other fractions. Anthraquinones, cardiac glycosides were not detected in these fractions, while saponin and flavonoids were only detected in WF-LMME and EA-LMME, respectively. Terpenoids were not detected in EA-LMME, while WF-LMME and CF-LMME had moderate-to-low levels of these phytoconstituents.

## 4. Discussion

The results obtained from this study showed that the fractions recovered from *L. micranthus *methanolic leaf extract elicited concentration-dependent (0–1.5 mg/mL) inhibition of norepinephrine and KCl-induced rat aorta smooth muscle contraction with the n-butanol fraction as the most active fraction followed by water (NBF-LMME), chloroform (CF-LMME) and ethylacetate (EAF-LMME) fractions. However, the relaxation effects of these fractions on the aorta smooth muscle were less pronounced against KCl-induced contraction compared to norepinephrine. Furthermore, mice given per orally 250 mg/kg bw of the n-butanol fraction also had the lowest levels of serum total cholesterol, LDL-cholesterol and triglycerides but highest serum nitric oxide level after 21 days of administrations compared to other fractions and the control. Norepinephrine is a pressor agent that mediates smooth muscle contraction via the *α*-adrenergic receptor whose activation leads to the activation of phospholipase C, which in turn produces inositol 1, 4, 5 triphosphate (IP_3_) and diacylglycerol (DAG). The consequence of this cascade is smooth muscle contraction arising from calcium mobilization from the intracellular store and membrane depolarization-dependent influx of calcium ion (Ca^2+^). The ionotropic response of the aorta smooth muscle to depolarizing high KCl (i.e., >30 mM) on the hand is mediated via voltage dependent Ca^2+^-channels which, opens to allow an influx of extracellular calcium ions (Ca^2+^) into smooth muscle cells for contractility [38]. 

The observed inhibition of contraction of the aorta ring in this study implies that our fractions are spasmolytic in action and antagonistic to the ionotropic effects of norepinephrine, an *α*-adrenoreceptor agonist. The EC_50_ and *R*_max_ values due to the n-butanol fraction against epinephrine-induced aorta contraction were found to be 0.65 mg/mL and 75.2%, respectively. Inhibition of rat aorta smooth muscle contraction due to KCl and phenylephrine another *α*-adrenergic receptor agent has also been reported for *L. ferrugineus* fractions and the n-butanol fraction was also found to be the most potent fraction but with a median effective concentration (EC_50_) of 38.4 ug/mL and maximum relaxation (*R*_max_) effect of 80.5% against phenylephrine induced contraction and EC_50_ of 726 ug/mL and *R*_max_ of 20.7% against KCl induced contraction [21]. In this study, for the water and chloroform fractions of *L.micranthus*, we obtained EC_50_ values of 1.18 mg/mL/>1.5 mg/mL and >1.5 mg/mL/>1.5 mg/mL and *R*_max_ values of 56.5%/10.4% and 35.9%/10.4% against PE/KCl induced contraction of the rat aorta smooth muscle, whereas EC_50_ values of 230 ug/mL/8.3 ug/mL and 217 ug/mL/933 ug/mL and *R*_max_ values of 53.7%/14.3% and 22.4%/6.6% were reported for these fractions from *L. ferrugineus*. Although by this result our fractions can be said to be less potent as spasmolytic agents compared to replica fractions from *L. ferrugineus*, baseline tension of 1.5 g was used to equilibrate our aorta ring preparation and maximum concentration of 1.5 mg/mL for each fraction was tested for spasmolytic effect in this study. Whereas in the study of *L. ferrugineus*, baseline tension of 1.0 g and maximum concentration of 3.0 mg/mL for each fraction were used [21]. Despite variations in these preload parameters, disparity in the antiionotropic potency between *L. ferrugineus* and *L. micranthus* may occur, since they are of a different species and may have different genetic background and phytochemistry coupled with the host from which they propagate as semiparasitic plants. Other plants with documented evidence for aorta smooth muscle vasorelaxation include ginger, the ethanolic extracts of *Morinda citrifola* root with an EC_50_ value of 1.65 mg/mL against phenylephrine induced rabbit aorta contraction [39], aqueous leaf extract of *Caesalpinia ferrea *[40], and methanolic leaf extracts of *Croton schiedeanus* Schlecht and *Calea glomerulata* with EC_50_s of 65 ug/mL and 71 ug/mL, respectively, against phenylephrine-induced rat aorta contraction [41]. The vasorelaxant mechanism adduced for these plants mimic those of antihypertensive drugs, and they include blockade of adrenergic receptors, and voltage dependent calcium channels, inhibition of calcium from intracellular stores, and opening of voltage; ATP and calcium activate K+ channels to cause hyperpolarization of smooth muscle membrane through further reduction of the resting membrane potential [39–41]. Similar to our findings, these plants also relaxed aorta in a concentration-dependent manner and some of them were found to inhibit calcium entry slowly and revert the contractility action mediated by this cation. Therefore, there is a high possibility that our *L. micranthus* fractions may use one or more of these mechanisms. However, there is a need to study the roles played by these spasmolytic mechanisms, particularly those that antagonize spasmogenic actions of norepinephrine. This include *α*-adrenoreceptor activation antagonized by adrenoreceptor antagonists such as papaverine, stimulation of phospholipase C activity that could be affected using a phosphodiesterase inhibitor, elevation in intracellular IP_3_ level, intracellular calcium dysregulation due to altered activity of endoplasmic reticulum Ca^2+^ ATPase and activation of voltage dependent membrane Calcium channel that could be antagonized by calcium channel blockers such as verapamil [38].

Another limitation of this study is that the aorta was not de-endotheliazed to study the role played by the endothelium since endothelium modulates vascular tone through the secretion of relaxing substances in which nitric oxide is a major component [7, 21, 39]. The endothelial nitric oxide release is in turn dependent on the activation of calcium-dependent nitric oxide synthase (NOS). The vasodilatory mechanism of *L. ferrugineus* fractions was purported to involve this pathway, while *Morinda citrifola* employed NO-independent pathways to mediate vasodilatory response in rabbit aorta smooth muscle [39].

Nevertheless, our observed higher serum NO levels but lower total cholesterol, LDL-cholesterol and TAG levels particularly in mice administered NBF-LMME and WF-LMME after 21 days of administration compared to the control provides an indication that these fractions contain antiartherogenic and vascular modulating substances. Arginase has been considered to be a diagnostic indicator for hypertension and other cardiovascular diseases [42]. Higher activity has been found to reduce arginine bioavailability as substrate for NOS in the endothelium, the pathologic consequences of which include increased vascular resistance, blood pressure and arthropathy [42, 43]. In this study, the NBF-LMME fraction was found to elicit a reduction in cardiac arginase by 11.7% of the control after 21 days of administration in our studied mice, while the water fraction caused 8.3% reduction in this enzyme activity. Therefore, our observation suggests arginase activity reduction as a possible antihypertensive mechanism of *L. micranthus* and further identify the n-butanol fraction as a better source antihypertensive principle in the plant. Cardiac arginase has been reported to be hemodynamically sensitive to blood pressure fluctuations and arginase inhibitors such as hydrazalazine and nor hydroxyl arginine have been demonstrated to hold promise as future antihypertensive agents courtesy of their abilities to cause reduction in arginase activity by up to 30% and arterial blood pressure by 30–35 mmHg, modulate arterial resistance and promote blood flow [42, 43]. It is important to note that cardiovascular diseases such as hypertension, arrythmias, angina pectoris, myocardial infarction, stroke, and left ventricular hypertrophy have become a major cause of morbidity and mortality in the world with increasing prevalence in developing countries [44]. Hypercholesterolemia, hypertriglyceridemia, elevated LDL-cholesterol and endothelial dysfunction have been recognized as risk factors of these diseases [45, 46]. Cholesterol and triglycerides are hydrophobic and are transported in systemic circulation through packaging in LDL-cholesterol, and very low density lipoprotein [46, 47]. Elevated levels of these lipids have been associated with age and consumption of high lipid diets [48]. These lipids are artherogenic because they enhance narrowing of the blood to retard blood flow and increase vascular resistance [48]. In severe cases, the flow of blood is completely blocked causing tissue ischaemia and necrosis [49]. The narrowing of the blood vessels is as a result of plaque formation involving artherogenic lipid modification by peroxidation and proliferation of underlying smooth cells and foam cell formation coupled with nitric oxide consumption to form reactive nitrogen species (RON) and release of adhesion molecules with vasoconstriction effect into the systemic circulation [49]. Therefore, diet modification, exercise, use of hypocholesterolemic and triglyceride-lowering drugs are key therapeutic options for the management of arthrosclerosis and other cardiovascular diseases [48].

 However, in this study we found no significant difference between the control and fraction-treated mice in the levels of HDL-cholesterol, creatinine and total protein suggesting that the antiartherogenic property of our fractions may not involve HDL-cholesterol elevation, and altered kidney and liver functions to modulate nitric oxide excretion or retention and enhance synthesis of secreted proteins.

Taken together, our findings indicate that *L. micranthus* possesses hypocholesterolemic, hypotriglyceridemic, and antioxidant substances in addition to its effect on nitric oxide metabolism. The results of this study have also validated the previous antihypertensive claims for this plant in Nigeria and South Africa [15–17]. 

Furthermore, we found steroids and terpenoids in moderate and high abundance in the n-butanol fraction, while tannins and other phenolic compounds were found to be mildly present in this fraction and the water fraction. These findings strongly suggest the possibility of involvement of these phytoconstituents in the observed vasorelaxant and antiartherogenic activity of our active fractions. There is also a possibility for noninvolvement of flavonoids in these activities since it was only detected in the ethylacetate fraction. Chromatographic peaks suggesting terpenoid abundance was found as the major bioactive substance that mediated the vasorelaxant and antihypertensive activity of the n-butanol fraction of *L. ferrugineus* methanolic extract [21], while tannins, Flavonoids, anthraquinones and steroids were implicated as vasorelaxant, antihypertensive and antidiabetic substances in *Morinda citrifola *[39]. In the work of Nishida and Satoh [50], Flavonoids and Terpenoids were implicated as vasorelaxant agents in *Ginkgo biloba*. These substances have also been detected in the crude aqueous and methanolic leaf extracts of *L. micranthus,* suggesting that by solvent-solvent fractionation, these substances are partitioned and concentrated in different fractions. Our findings, thus, indicate that the bioactive substances involved in the cardiovascular activity of *L. micranthus* are polar in nature. However, the possibility of noninvolvement of flavonoids needs further confirmation with more sensitive methods.

Based on the results obtained so far in this study, we conclude that the previously reported antihypertensive activity of *L. micranthus* mechanistically involve vasorelaxation, elevation of serum nitric oxide, and antiartherogenic effect against systemic triglyceride and cholesterol levels in mice with terpenoids, steroids, and tannins as potential bioactive substances mediating these mechanisms.

## Figures and Tables

**Table 1:** Changes in serum lipid levels in mice orally administered with the various fractions recovered from *Loranthus micranthus* methanolic leaf extract for 21 days.

			Treatment group			
		Control	NBF-LMME	WF-LMME	CF-LMME	EAF-LMME
Parameter	Day 0	Day 21	Day 21	Day 21	Day 21	Day 21
T-CHOL, mg/dL	88.9 ± 0.8^a^	114.3 ± 3.5^b^	91.9 ± 0.4^a^	95.5 ± 2.8^c^	107.1 ± 0.7^b^	108.9 ± 2.8^b^
% Δ	—	(28.6)	(3.4)	(7.4)	(20.5)	(22.5)
LDL-CHOL, mg/dL	18.2 ± 0.3^a^	42.2 ± 4.3^b^	23.7 ± 0.9^c^	25.0 ± 3.4^c^	35.3 ± 0.6^d^	36.1 ± 3.1^d^
% Δ	—	(131.9)	(30.2)	(37.4)	(94.0)	(98.4)
TAG, mg/dL	147.3 ± 2.5^a^	152.8 ± 0.6^b^	138.5 ± 3.4^c^	148.1 ± 4.0^a^	151.9 ± 1.2^b^	152.2 ± 2.7^b^
% Δ	—	(3.7)	(−6.0)	(0.5)	(3.1)	(3.3)
HDL-CHOL, mg/dL	41.3 ± 0.9^a^	41.6 ± 0.7^a^	41.0 ± 0.4^a^	40.8 ± 0.7^a^	41.5 ± 0.5^a^	40.6 ± 0.2^a^
% Δ	—	(0.7)	(−0.7)	(−1.2)	(0.5)	(−1.7)

Data are mean ±SEM of triplicate assays. Figures in each row with different superscript letters (a, b, c, d) are significantly different (*P* < 0.05). Figures in parentheses represent percentage change (%Δ) in day 21 lipid level compared to day 0 (increase = +; decrease = −). This reflects the multiple range test statistics used to compare the mean values of variables measured across the *Loranthus micrathus* fraction groups.

**Table 2:** Changes in cardiac arginase activity and serum total protein, creatinine, and nitric oxide in mice orally administered with the various fractions recovered from *Loranthus micranthus* methanolic leaf extract for 21 days.

			Treatment group			
		Control	NBF-LMME	WF-LMME	CF-LMME	EAF-LMME
Parameter	Day 0	Day 21	Day 21	Day 21	Day 21	Day 21
Total Protein, g/dL	5.14 ± 0.05^a^	5.17 ± 0.04^a^	5.21 ± 0.06^a^	5.14 ± 0.08^a^	5.22 ± 0.02^a^	5.18 ± 0.05^a^
% Δ	—	(1.6)	(1.2)	(0)	(1.5)	(1.7)
Creatinine, mg/dL	0.34 ± 0.04^a^	0.37 ± 0.03^a^	0.32 ± 0.04^a^	0.40 ± 0.07^a^	0.31 ± 0.07^a^	0.38 ± 0.03^a^
% Δ	—	(8.8)	(−5.9)	(17.6)	(−8.8)	(11.2)
NO, umole/L	14.9 ± 0.1^a^	14.5 ± 0.6^a^	23.1 ± 1.3^b^	18.2 ± 1.5^c^	16.1 ± 1.8^a^	15.3 ± 0.2^a^
% Δ	—	(3.7)	(55.0)	(22.1)	(8.1)	(2.7)
Arginase, unit/mg tissue	98.3 ± 3.8^a^	101.8 ± 1.7^b^	86.8 ± 3.5^c^	90.1 ± 1.5^d^	95.7 ± 1.5^a^	97.1 ± 3.4^a^
% Δ	—	(3.6)	(−11.7)	(−8.3)	(−2.6)	(−1.2)

Data are mean ±SEM of triplicate assays. Figures in each row with different superscript letters (a, b, c, d) are significantly different (*P* < 0.05). Figures in parentheses represent percentage change (%Δ) in day 21 lipid level compared to day 0 (increase = +; decrease = −). This reflects the multiple range test statistics used to compare the mean values of variables measured across the *Loranthus micrathus* fraction groups.

**Table 3:** Phytochemical screening of fractions recovered from *Loranthus micranthus* methanolic leaf extract.

	Fractions			
Phytoconstituent	NBF-LMME	WF-LMME	CF-LMME	EA-LMME
Flavonoids	−	−	−	+
Terpenoids	+++	++	+	−
Tannins	+	+	++	+
Phenolics	+	+	++	+
Saponin	−	+	−	−
Anthraquinones	−	−	−	−
Cardiac glycosides	−	−	−	−
Steroids	++	+	−	−
Reducing sugars	+	++	+	+

Abundance indicator: +++ = Highly present; ++ = Moderately present; + = Lowly present; − = Absent.
